# Using an Innovative Data Warehouse to Determine Nurse Staffing Indicators Associated With Medication Errors: A Correlational Study

**DOI:** 10.1155/jonm/6958869

**Published:** 2026-07-23

**Authors:** Kahina Bensaadi, José Côté, Gabrielle Chicoine, Michael Yu, David Boucher-Roy, Carl-Ardy Dubois

**Affiliations:** ^1^ Research Centre, Centre Hospitalier de l’Université de Montréal, 850 Rue Saint-Denis, Montréal Quebec, H2X 0A9, Canada, chumontreal.qc.ca; ^2^ Faculty of Pharmacy, Université de Montréal, C.P. 6128 Succursale Centre-Ville, Montréal Quebec, H3C 3J7, Canada, umontreal.ca; ^3^ Faculty of Nursing, Université de Montreal, C.P. 6128 Succursale Centre-Ville, Montréal Quebec, H3C 3J7, Canada, umontreal.ca; ^4^ Li Ka Shing Knowledge Institute, St. Michael’s Hospital, 30 Bond Street, Toronto Ontario, M5B 1W8, Canada, stmichaelshospital.com; ^5^ School of Public Health, Université de Montréal, C.P. 6128 Succursale Centre-Ville, Montréal Quebec, H3C 3J7, Canada, umontreal.ca

**Keywords:** healthcare, hospital, medication errors, nurses, nursing staff, personnel staffing, quality indicators

## Abstract

**Introduction:**

A growing body of literature suggests that medication errors (MEs) result from a complex interaction of factors related to inadequate medication management systems or from human factors and staffing shortages. Research has identified several significant associations between MEs and nurse staffing indicators, but the evidence is moderate and varies considerably across studies. Most of this research has been conducted in the United States. Few studies have been undertaken in Canada.

**Objective:**

The purpose of this study was to demonstrate the feasibility of using an innovative data warehouse to assess the association of nurse staffing indicators with MEs in a Canadian context.

**Methods:**

A correlational design was used to examine retrospective data (2019–2021) from an innovative data warehouse (LEPSI) located in a university hospital center in a metropolitan area. Specifically, 94,145 patient files, 8368 employee profiles, and 2813 MEs were analyzed. A generalized linear mixed model was used to model the relationship between nurse staffing indicators and MEs, with confounding variables controlled.

**Results:**

A significant association emerged between lower odds of ME occurrence and, respectively, higher ratios of clinical nurse (bachelor’s degree) and nurse technician (professional/vocational diploma) hours to overall care team member hours (OR: 0.46, 95% CI: 0.32–0.66, *p* < 0.001) and higher ratios of clinical nurse hours to care team hours (OR: 0.41, 95% CI: 0.31–0.54, *p* < 0.001). No evidence emerged to support an association between MEs and, respectively, higher ratios of overall care team hours to overall patient hours (OR: 1.01, 95% CI: 0.83–1.24, *p* = 0.890) and higher ratios of care team overtime hours to care team hours (OR: 0.71, 95% CI: 0.47–1.06, *p* = 0.097).

**Conclusions:**

Results proved consistent with the literature, thus confirming the feasibility of using the LEPSI data warehouse for nursing performance measurement. The use of a data warehouse to assess the association between nurse staffing indicators and MEs constitutes a promising approach for generating evidence to inform decision making related to patient safety.

## 1. Introduction

The Institute for Safe Medication Practices Canada [1] defines a medication error (ME), also referred to as a medication incident, as follows.

Any preventable event that may cause or lead to inappropriate medication use or patient harm while the medication is in the control of the healthcare professional, patient, or consumer. Medication incidents may be related to professional practice, drug products, procedures, and systems and include prescribing, order communication, product labelling/packaging/nomenclature, compounding, dispensing, distribution, administration, education, monitoring, and use.

According to the World Health Organization (WHO), MEs cost US$42 billion a year worldwide [2]. A growing body of evidence shows that MEs result from the complex interaction of factors in the clinical environment rather than from mere negligence within isolated professional practices [2–4]. They often occur when inadequate medication management systems or human factors such as fatigue, poor environmental conditions, and staffing shortages affect how medication is prescribed, documented, dispensed, administered, and monitored.

Nursing care teams, which include registered nurses, licensed practical nurses, and nursing aides/unlicensed assistive personnel, are on the front line of healthcare systems. Nurses are at the end of the drug administration chain and play a key role in ensuring that the right patient receives the right drug, in the right dose, at the right time, via the right route [5]. This is why a number of works have included MEs among nursing‐sensitive outcomes, that is, outcomes that are influenced by the work of nurses, even though nursing is not exclusively responsible for them. A deeper understanding of the relationship between nurse staffing indicators and MEs could contribute to the development and implementation of policies that prevent and reduce MEs.

Several systematic reviews have focused on the relationship between staffing indicators and MEs among other patient outcomes such as falls, pressure ulcers, predischarge counseling, nosocomial infections, length of hospital stay, readmission, and unsuccessful resuscitation [6–8]. Blume et al. [9] examined 15 knowledge syntheses published from 2007 to 2018, including seven systematic reviews of nurse staffing indicators affecting MEs (among other patient outcomes) in intensive care unit settings, including care team composition, work schedules (overtime and long working hours), and resource intensity (patient–nurse ratios). They concluded that the evidence supporting the relationship between MEs and the various nurse staffing indicators investigated was moderate. A recently conducted knowledge synthesis—not included in the Blume et al. umbrella review—further suggested that care team composition and resource intensity influenced MEs. For example, in their review of 39 studies, Oner et al. [7] found that five studies that examined predictors of MEs reported a significant positive relationship between ME occurrence and ratio of patients to total nurses and a significant negative relationship between ME occurrence and proportion of registered nurses in the nursing staff. In their integrative review of the influence of nurse characteristics on MEs covering 19 studies, Kerari and Innab [10] reported that of the seven studies included that examined nurse education level as an ME predictor, five found that fewer MEs occurred when nurse care teams were composed of a greater number of nurses with a bachelor’s degree. In their systematic review of 63 studies that examined the composition of care teams, Twigg et al. [8] found that three of the seven studies included that assessed MEs observed no statistically significant differences across compositional variations, whereas the other four reported a significant decrease in MEs when the proportion of registered nurses in the team increased. Regarding work schedules, Bae [6] examined 22 studies published from 2000 to 2019 and found that of the five studies included that assessed MEs, none observed a significant relationship between voluntary or mandatory overtime and MEs. However, numerous significant associations have been noted between MEs and care team composition and resource intensity [7, 10–13]. The existing literature shows a great variability across studies in terms of evaluation methods, measurement approaches, and measures. This makes it difficult to compare studies and draw any universal conclusions based on their results.

Assessing whether a relationship exists between nurse staffing and patient outcomes depends on how reliable the measurement of these indicators is. According to the nursing care performance framework (NCPF) [14], nursing care performance assessment relies on three interrelated dimensions: (1) acquisition, deployment, and maintenance of nursing resources; (2) transformation of resources into nursing services; and (3) production of changes in the condition of patients. However, the identification of robust measures to assess nursing care performance is hindered by incomplete or nonstandardized data entry at source, burdensome documentation, and costly data collection. Also, once entered in a database, data must be retrieved and organized before analysis. The cost of doing so has had a dissuasive effect on the development of robust and comparable measures. To tackle these challenges, we developed LEPSI (*Laboratoire de développement et d’Expérimentation en contexte réel d’un système de gestion de la Performance des Soins Infirmiers*), an innovative data warehouse and lab platform, to develop a nursing care performance data management system for the purpose of conducting real‐world research. LEPSI makes it possible to collect clinical and administrative data to measure nursing care quality indicators in a homogenous, standardized, and reliable manner.

### 1.1. Objective

We sought to assess the feasibility of using the LEPSI data warehouse to identify nurse staffing indicators associated with MEs in a Canadian university hospital.

A hypothesis was formulated for each of the three domains of indicators: quantity and intensity of human care resources; nursing care team composition; and work schedules (overtime).

 Hypothesis 1. The higher the intensity of human care resources, the lower the MEs.
 Hypothesis 2. The higher the ratio of clinical nurses (bachelor’s degree) and nurse technicians (professional/vocational diploma) to care team and the higher the ratio of clinical nurses to care team, the lower the MEs.
 Hypothesis 3. The higher the ratio of overtime hours to care team hours, the higher the MEs.

Benchmarking the corresponding results against the findings of prior research that utilized comparable indicators would then provide a measure of the match and, thus, of the feasibility of using the LEPSI to measure nursing performance.

## 2. Methods

A correlational research design was used to analyze the LEPSI data from 2019 to 2021. The study was developed based on the NCPF, a comprehensive theoretical model for implementing indicators and their interrelations as tools for care performance assessment [14–16]. The Strengthening the Reporting of Observational Studies in Epidemiology (STROBE) Statement [17] served as a reporting guide (Appendix 1).

### 2.1. Setting and Participants

The study was conducted in a large, quaternary, academic hospital center located in a metropolitan area in Eastern Canada (Montreal, Quebec). The LEPSI was developed and implemented by a multidisciplinary team of professionals with complementary expertise, which included researchers, biostatisticians, healthcare managers, and healthcare professionals.

Data regarding the human care resources used in the course of adult inpatient hospital stays of at least 24 h were extracted from the data warehouse. Ambulatory units (ER and outpatient clinics) were excluded. Care teams were composed of registered nurses (clinical nurses and nurse technicians), licensed practical nurses, and unlicensed assistive personnel (or personal support workers). Clinical nurses held a bachelor’s degree, nurse technicians held a postsecondary professional/vocational diploma, licensed practical nurses held a high school diploma, and unlicensed assistive personnel held a Skills Training Certificate.

### 2.2. Variables

Variables were collected for each work shift. The work shift was the unit of analysis.

#### 2.2.1. Nurse Staffing Indicators

Drawing on the NCPF [14] and in light of the objective of this study, we assessed and measured three main nurse staffing indicators:

1. Quantity and intensity of human care resources were measured by the ratio of care team hours to patient hours.
2. Nursing care team composition was measured by the ratio of clinical nurse and nurse technician hours to care team hours and by the ratio of clinical nurse hours to care team hours.
3. Work schedules were measured by the ratio of care team overtime hours to care team hours.

#### 2.2.2. Medication Errors

MEs were self‐reported by nursing care teams as part of the provincial error monitoring process. Under this system, nurses are asked to report MEs according to where they occur in the medication use process, that is, at time of medication supply, order issue, medication management, or processing of medical prescriptions on the care unit. Only MEs potentially attributable to nursing staff were considered in this study, that is, MEs that occurred at time of medication management and processing of medical prescriptions on the care unit.

#### 2.2.3. Patient Variables

The following patient sociodemographic and clinical variables were collected: age, sex, reason for hospital admission, presence of comorbidity, medication, length of hospital stay, and occurrence of MEs.

#### 2.2.4. Confounding Variables

The following confounding factors that could potentially influence the relationship between nurse staffing indicators and MEs were controlled: work shift (day, evening or night), care unit, care unit type, weighted mean Charlson Comorbidity Index (CCI) score, weighted mean age of patients, and patient turnover rate (see Appendix 2).

### 2.3. Data Sources

LEPSI data were filtered for hospital stays from 2019 to 2021, in 31 adult inpatient units, lasting over 24 h, at the Centre Hospitalier de l’Université de Montréal (CHUM). The data covered human care resources (registered nurses, licensed practical nurses, and unlicensed assistive personnel), patients (hospital stays and sociodemographic and clinical characteristics), and adverse events, including MEs. The warehouse source data came from different systems: staffing data from the human resources management (GRH) system, ME data from the patient care and services safety information system (SISSS), and patient data from the hospital admission, discharge, and transfer (ADT) system and from the electronic medical system archives named OACIS (Appendix 3).

### 2.4. Sample Size

The data covered a total of 94,145 patient files, 8368 employee identification numbers (ID), and 2813 reported MEs.

### 2.5. Statistical Methods

We used a generalized linear mixed model (GLMM) with a logit link function to model the relationship between each nurse staffing indicator and the occurrence of at least 1 ME on a work shift. The effect of each nurse staffing indicator was examined independently of the other indicators (i.e., one nurse staffing indicator per model) on account of the high collinearity between them. Each model included the variable of interest, a nurse staffing indicator as a fixed effect, and the confounding variables as either a fixed effect (patient turnover rate, weighted mean CCI score of patients on unit during work shift by patient hours, and weighted mean age of patients on unit during work shift by patient hours) or as a random effect (care unit nested in care unit type), according to their nature. The global effect of the nurse staffing indicators, the intraclass correlation coefficient, and the effect of confounding factors in the association between MEs and nurse staffing indicators were extracted from the models. The association between ME and staffing indicator was assessed via odds ratio (OR) with a 95% confidence interval (CI). The significance level was set at 5%, and no adjustments were made for multiple comparisons.

### 2.6. Ethical Considerations

The study and the development of the LEPSI received ethics approval from the CHUM Research Ethics Committee (#22.124 and #19.094).

## 3. Results

### 3.1. Descriptive Results

**FIGURE 1:**
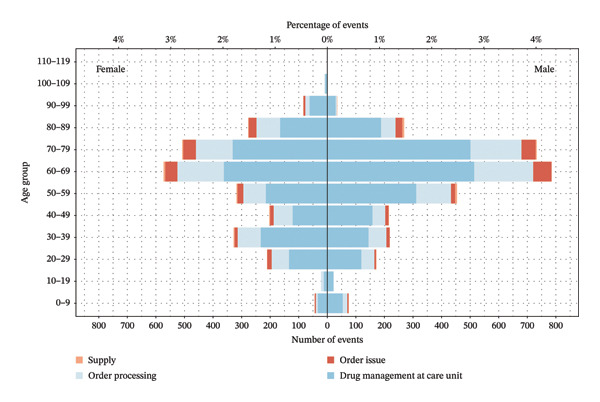
Distribution of absolute and relative number of MEs by patient age, gender, and medication use process at care unit.

The vast majority of shifts had no MEs (96.71%), a few had one (2.67%), and even fewer had more than one (0.62%) (see Table 1). MEs occurred most frequently during medication management and the processing of medical prescriptions on the care unit. They were more frequent with male patients and with patients 60–79 years of age (see Figure 1). Even though more than 1 ME could occur per shift, the distribution of errors was extremely right‐skewed: Shifts with multiple errors proved relatively rare. Because the vast majority of shifts in which MEs occurred registered the occurrence of only 1 ME, we chose to treat MEs as a binary variable, that is, no ME or at least one. MEs related to medication management and prescription processing on the care unit occurred most often on day (3.39%) and evening (3.34%) shifts and less often on night shifts (3.14%). On average, 50% of the nursing staff was composed of clinician and technician nurses. While the rate of licensed practical nurses varied across units, unlicensed assistive personnel made up 30% of the nursing staff on most of the care units (data not shown).

**TABLE 1:** Distribution of number of ME incidents per work shift.

Number of MEs on shift	*n*/*N* (%)
0	82,673/85,486 (96.71%)
1	2284/85,486 (2.67%)
2	394/85,486 (0.46%)
3	77/85,486 (0.09%)
4	26/85,486 (0.03%)
5	16/85,486 (0.02%)
6	6/85,486 (0.01%)
7	4/85,486 (0.005%)
8	1/85,486 (0.001%)
9	2/85,486 (0.002%)
10	2/85,486 (0.002%)
11	1/85,486 (0.001%)

### 3.2. Nurse Staffing Indicators and MEs

Staffing indicators were modelized separately given the high collinearity between them. Therefore, each staffing indicator was evaluated in isolation and reported effects were not mutually adjusted. Results regarding Hypothesis 1 are presented in Table 2, those regarding Hypothesis 2 in Tables 3 and 4, and those regarding Hypothesis 3 in Table 5. Where Hypothesis 1 is concerned, we found no clear association between ratio of care team hours to patient hours and MEs. Regarding Hypothesis 2, a higher ratio of clinical nurse and nurse technician hours to care team hours and a higher ratio of clinical nurse hours to care team hours significantly lowered the odds of an ME occurring. As for Hypothesis 3, a higher ratio of care team overtime hours to care team hours did not significantly change the odds of an ME occurring.

**TABLE 2:** Global effect of ratio of care team hours to patient hours on at least one ME occurring on work shift.

	Coefficient (standard error)	Odds ratio [95% CI]	*p* value
Care team hours/patient hours	0.01 (0.10)	1.01 [0.83–1.24]	0.890
Work shift type: evening^∗^	−0.09 (0.06)	0.91 [0.81–1.02]	0.099
Work shift type: night^∗^	−0.03 (0.05)	0.97 [0.88–1.07]	0.566
Patient turnover rate	0.00 (0.00)	1.00 [0.99–1.00]	0.572
Mean CCI score of patients on work shift	−0.05 (0.04)	0.95 [0.89–1.02]	0.146
Mean age of patients on work shift	0.00 (0.00)	1.00 [0.99–1.01]	0.498

Abbreviations: CCI, Charlson Comorbidity Index; CI, confidence interval.

^∗^Day shift was the reference category.

**TABLE 3:** Global effect of ratio of clinical nurse and nurse technician hours to care team hours on at least one ME occurring on work shift.

	Coefficient (standard error)	Odds ratio [95% CI]	*p* value
Clinical nurse and nurse technician hours/care team hours	−0.78 (0.19)	0.46 [0.32−0.66]	< 0.001
Work shift type: evening^∗^	−0.10 (0.06)	0.90 [0.81−1.01]	0.063
Work shift type: night^∗^	−0.02 (0.05)	0.98 [0.89−1.08]	0.661
Patient turnover rate	0.00 (0.00)	1.00 [0.99−1.00]	0.726
Mean CCI score of patients on work shift	−0.04 (0.04)	0.96 [0.90−1.03]	0.308
Mean age of patients on work shift	0.00 (0.00)	1.00 [0.99–1.01]	0.550

Abbreviations: CCI, Charlson Comorbidity Index; CI, confidence interval.

^∗^Day shift was the reference category.

**TABLE 4:** Global effect of ratio of clinical nurse hours to care team hours on at least one ME occurring on work shift.

	Coefficient (standard error)	Odds ratio [95% CI]	*p* value
Clinical nurse hours/care team hours	−0.89 (0.14)	0.41 [0.31–0.54]	< 0.001
Work shift type: evening^∗^	−0.13 (0.06)	0.88 [0.79–0.98]	0.022
Work shift type: night^∗^	−0.03 (0.05)	0.97 [0.88–1.07]	0.575
Patient turnover rate	0.00 (0.00)	1.00 [0.99–1.00]	0.890
Mean CCI score of patients on work shift	−0.05 (0.04)	0.95 [0.89–1.02]	0.179
Mean age of patients on work shift	0.00 (0.00)	1.00 [0.99–1.01]	0.661

Abbreviations: CCI, Charlson Comorbidity Index; CI, confidence interval.

^∗^Day shift was the reference category.

**TABLE 5:** Global effect of ratio of care team overtime (OT) hours to care team hours on at least one ME occurring on work shifts.

	Coefficient (standard error)	Odds ratio [95% CI]	*p* value
Care team OT hours/care team hours	−0.35 (0.21)	0.71 [0.47−1.06]	0.097
Work shift type: evening^∗^	−0.09 (0.06)	0.92 [0.82−1.02]	0.110
Work shift type: night^∗^	−0.02 (0.05)	0.98 [0.89−1.07]	0.624
Patient turnover rate	0.00 (0.00)	1.00 [0.99−1.00]	0.522
Mean CCI score of patients on work shift	−0.05 (0.04)	0.95 [0.89−1.02]	0.148
Mean age of patients on work shift	0.00 (0.00)	1.00 [0.99−1.01]	0.521

Abbreviations: CCI, Charlson Comorbidity Index; CI, confidence interval.

^∗^Day shift was the reference category.

## 4. Discussion

### 4.1. Main Findings

The aim of this study was to test the feasibility of using the LEPSI data warehouse to identify nurse staffing indicators associated with MEs in real‐world practice settings. Using modeling to examine the association between three nurse staffing indicators and MEs, we observed that a higher proportion of nursing staff with a higher education level in the care team composition was associated with a significant reduction in ME events. However, the results concerning indicators related to quantity and intensity of human resources and to work schedules revealed no significant association with MEs. How do these results stack up against those reported in the literature?

Our results showed that care team composition was associated with ME occurrence. This is in line with results reported by authors who examined the relationship between care team composition and a host of patient outcomes, including MEs [7, 11], and with those reported by authors who examined the influence of nurse education level on MEs [10]. In the systematic review by Oner et al. [7], all the studies reported a significant relationship where fewer medication administration errors occurred when teams comprised a higher proportion of registered nurses and when the ratio of registered nurses to patients was higher. In their integrative review, Kerari and Innab [10] observed that an increase in resources with a lower education level (e.g., Associate Degree in Nursing) led to an increase in MEs and that these resources were more likely than nurse clinicians to be responsible for MEs when MEs occurred (68.3% vs. 26.2%). The knowledge, attitude, skills, and clinical acumen conferred by the level of education completed by clinical nurses (i.e., university degree), as opposed to what is acquired over the course of a vocational–technical degree, could potentially play a role in nurses’ ability to manage medication and anticipate omissions and errors.

Only two studies considered in systematic reviews found a statistically significant relationship between quantity and intensity of human resources and ME events. Our results are consistent with the vast majority of studies that did not observe such an association [7, 12]. In this regard, an earlier Canadian study showed that increasing resource intensity alone was not sufficient to improve quality of care and patient outcomes, including MEs. Dubois et al. explored the matter from the angle of different nursing performance models: basic/innovative professional models and basic/adaptive functional models. Professional models were those that employed more nursing workers with higher formal education. They were characterized both by a higher proportion of care hours provided by RNs and by nurses’ perception of greater support for their professional practice. Functional models, instead, depended more on less educated staff, including licensed practical nurses and unregulated assistive staff, to deliver nursing services. They were characterized both by a lower proportion of care hours provided by RNs and by nurses’ perception that the practice environment was less supportive of a “professionalized” approach to their work. Specifically, Dubois et al. found that MEs were significantly lower by 25%–52% in the two professional staffing models that focused on employing resources with a higher level of education, compared with the functional staffing models that focused on employing more resources with varying levels of education. What is more, the frequency of consequential MEs was about 40% lower in units with the basic functional model (i.e., focused on the use of more resources but less trained resources) than in those with the adaptive functional model (i.e., focused on more staffing intensity). The relatively poor results of the “adaptive functional” model, which employed more resources per patient than did the “basic functional” model, suggested that, although staffing intensity might be important, it might not be sufficient on its own to be associated with improved outcomes. Beyond staffing intensity, a combination of factors, including care team composition, support for professional practice, capacity for innovation, and nurses’ scope of action, explained the differences between the various types of nursing organizational structures [14].

Similarly, our results also showed no statistically significant relationship between work schedules (i.e., overtime) and MEs. This result may seem surprising given that fatigue has been associated with reduced cognitive performance and a lack of attention and vigilance [18, 19] and therefore one might expect overtime to cause more MEs. To be sure, there is evidence supporting an association between working more than 40 h per week and increased frequency of ME events. However, in a recent systematic review, Bae [6] found that none of the primary studies included had reported a statistically significant relationship between MEs and overtime, whether mandatory or voluntary. Care delivery interruptions are probably an element to consider when analyzing and interpreting overtime as an indicator of care quality. While overtime has been associated with fatigue and loss of concentration and alertness, working many hours in a row can reduce staff turnover and, by that token, avoid care delivery interruptions. In other words, these interruptions may play a major role in the occurrence of MEs. In the literature, care delivery interruptions have been described as having a significant impact on medication management, mainly at the time of medication administration rather than during other nursing activities. They have also been described as a factor leading to abrupt procedure terminations responsible for clinical errors [20, 21]. It may be hypothesized, then, that while overtime increases the risk of MEs, overtime also translates into fewer care delivery interruptions, and this could moderate the effect on ME occurrence.

Finally, the congruence of our results with the state of knowledge in the field confirms the feasibility of using the LEPSI to measure nursing care performance. Also, our study contributes to the advancement of knowledge in the field by adding to the pool of evidence supporting the association between various nurse staffing indicators and the probability of MEs occurring, for which the level of evidence was considered to be no more than moderate. This data warehouse was developed to serve two purposes. The first is to feed researchers high quality interoperable data available in quasi–real time. The second is to feed an operational dashboard codesigned with nurse managers to display nursing‐sensitive performance indicators. It is hoped that the dashboard will provide decision makers with valuable performance indicators to help them make informed decisions.

### 4.2. Strengths and Limitations

To our knowledge, this is the first study to use data from an innovative data warehouse, LEPSI, to identify nurse staffing indicators associated with MEs. We demonstrated the feasibility of using clinical‐administrative databases to measure the strength of associations between different nurse staffing indicators and MEs. The study shows how using a data warehouse can overcome the considerable constraints (e.g., difficulty producing robust measures to assess nursing care performance, incomplete or nonstandardized data entry at source, burdensome documentation, and costly data collection) that hinder the measurement of care quality and the prevention of adverse events, including MEs. Importantly, using an established conceptual framework for nursing care performance to select and synthesize standardized measures related to MEs enhanced the quality and robustness of our findings and facilitated their comparison with other studies.

Our study also highlighted certain challenges regarding the use of data warehouses. For one thing, with clinical‐administrative databases, it is only possible to consider electronically coded data. In our case, for example, it was impossible to measure time spent at patient’s bedside, as this information is not captured in the human resources management system. Moreover, the information on MEs available in the database was self‐reported as part of the provincial error monitoring process, which means that MEs may be underestimated. Also, MEs are considered rare events: Only 3.3% of the shifts considered in our model had at least one ME. This tends to affect the precision of estimations. What is more, data from different source systems must be transformed before they can be used together, and these transformations rest on assumptions that impact the interpretability of the data. For example, in calculating ratios regarding quantity and intensity of resources (e.g., ratio of care team hours to patient hours), we deducted time for legally permitted breaks on the assumption that resources availed themselves of this time in full, though they may not have in fact.

Some factors limit the generalizability and clinical applicability of our results. First, our models captured global effects that might have varied across the individual units. In addition, the data in our study were derived from a single quaternary university hospital center in Canada, which may have resources and policies of its own that impact MEs—resources and policies that differ from those in other hospitals. Additionally, use of the work shift as the unit of analysis limits, to some extent, the possibility of comparing our findings with the existing literature (e.g., human resource intensity measured by the ratio of nurse‐hours to patient‐hours versus nursing hours per patient day). Another limitation of this study is that both exposures (e.g., staffing intensity, overtime, and turnover) and outcomes (MEs) were aggregated at the shift level. This may obscure short‐term peak‐risk periods within shifts. Brief episodes of understaffing, workflow interruptions, and fatigue‐related risk are therefore not fully captured by the models. While this approach is common in the nurse staffing literature and aligns with the managerial level at which staffing decisions are made, it introduces temporal smoothing that may attenuate observed associations, particularly for staffing intensity and overtime. Finally, the results of this study must be understood and interpreted as associations derived from the examination of a retrospective cohort of data. Ultimately, prospective interventional studies are needed to confirm the reported associations between nurse staffing indicators and patient outcomes such as MEs.

## 5. Conclusions

We showed in this study that a newly developed data warehouse, LEPSI, could be used to collect and rapidly access homogenous, standardized, interoperable, and reliable clinical‐administrative data to assess nursing performance. Our results are consistent with the literature, suggesting that there is still insufficient evidence to support the association between increased intensity of resources and occurrence of MEs. Increasing the quality of resources, however, did prove to be associated with ME occurrence.

The ability of such databases to consistently reflect research findings suggests that they might also be used by hospitals and researchers to help assess new staffing models dynamically. This could lead to better‐informed decision‐making in terms of staffing and, in turn, improve patient care, which is all the more important in the current global context of staff shortages.

## Funding

This project was funded by the Canada Foundation for Innovation (Grant number 36910).

## Conflicts of Interest

The authors declare no conflicts of interest.

## Supporting Information

Additional supporting information can be found online in the Supporting Information section.

## Supporting information


**Supporting Information 1** Appendix 1. Completed STROBE checklist. This checklist was used to ensure the transparent and standardized reporting of this observational correlational study, in line with the Strengthening the Reporting of Observational Studies in Epidemiology (STROBE) guidelines.

**Supporting Information 2** Appendix 2. List of controlled confounding variables. This appendix details the confounding factors that were statistically adjusted for in the analysis, including work shift, care unit, unit type, Charlson Comorbidity Index (CCI), mean patient age, and patient turnover rate.

**Supporting Information 3** Appendix 3. Description of data sources. This appendix provides details on the data extracted from the LEPSI data warehouse, covering hospital stays from 2019 to 2021 across 31 adult inpatient units at the hospital. It includes descriptions of datasets related to nurse staffing, patient characteristics, and medication errors, as well as the specific hospital information systems from which the data were drawn (GRH, SISSS, ADT, and OACIS).

## Data Availability

The data that support the findings of this study are available from the corresponding author upon reasonable request.
