# Supplementary material for: Using an Innovative Data Warehouse to Determine Nurse Staffing Indicators Associated With Medication Errors: A Correlational Study
Source: J Nurs Manag. 2026 Jul 23;2026:6958869. doi: 10.1155/jonm/6958869 (PMC13393065; doi:10.1155/jonm/6958869)
Supplement: Supplementary file 2 — Supporting Information 2 Appendix 2. List of controlled confounding variables. This appendix details the confounding factors that were statistically adjusted for in the analysis, including work shift, care unit, unit type, Charlson Comorbidity Index (CCI), mean patient age, and patient turnover rate. [file JONM-2026-6958869-s002.pdf]

Appendix 2 – List of controlled confounding variables

We controlled for the following confounding factors that could have had an influence on the relationship between staffing and MEs:

Work shift type: The work shift was the unit of analysis used in the models of ME and staffing. Individual shifts were defined by: (1) date, (2) type, and (3) care unit. All shifts dated January 6, 2019, to December 5, 2021, were included. Three shift types were defined in line with the hospitals most common shift schedules: night (0:00-8:00), day (8:00-16:00), and evening (16:00-24:00). Day shifts were used as the base shifts in the statistical model.

Care unit and care unit type: The care unit referred to a collection of beds, typically collocated, under the care of the same nursing team. Each care unit offered care adapted to a specific type of patient. All hospitalization units that could have patients hospitalized for 24 hours or more were included. Ambulatory units (emergency, outpatient clinics) and hospitalization units of less than 24 hours and neonatal units were excluded. Of the 31 units included, some were grouped together (e.g., some units were separated in the ADT system but not in the human resources system). Consequently, analyses were done on 23 care or grouped units characterized by specific staffing and patient categories. The units were also categorized by type of care provided.

Weighted mean Charlson Comorbidity Index (CCI) score of patients present on a specific shift. The CCI is a measure of patient acuity using a prognostic taxonomy of patient comorbidities based on ICD-9 and ICD-10 diagnosis codes, which alone or in combination may modify a patient's short-term mortality risk (Charlson et al., 1987). The CCI score for each patient was calculated from archive data. A correspondence between the ICD-9 codes used in Quebec (a list of codes adapted for Quebec was used by archivists) and ICD-10 codes was used. This correspondence was based on the work of Simard et al. (2018). The weights assigned to each comorbidity were taken from the work of Lix et al. (2016), page 72. In order to capture the acuity of patients present on a shift, a weighted average of their CCI scores was used. Weights corresponded to the number of hours spent by each patient on the shift.

Weighted mean age of patients present on a specific shift. The weights corresponded to the number of hours spent by each patient on the shift.
