# Supplementary material for: Using an Innovative Data Warehouse to Determine Nurse Staffing Indicators Associated With Medication Errors: A Correlational Study
Source: J Nurs Manag. 2026 Jul 23;2026:6958869. doi: 10.1155/jonm/6958869 (PMC13393065; doi:10.1155/jonm/6958869)
Supplement: Supplementary file 3 — Supporting Information 3 Appendix 3. Description of data sources. This appendix provides details on the data extracted from the LEPSI data warehouse, covering hospital stays from 2019 to 2021 across 31 adult inpatient units at the hospital. It includes descriptions of datasets related to nurse staffing, patient characteristics, and medication errors, as well as the specific hospital information systems from which the data were drawn (GRH, SISSS, ADT, and OACIS). [file JONM-2026-6958869-s003.pdf]

Appendix 3– Description of data sources

Employee data	*Patient data*	*Care unit data*
Employee ID<br>***HRM***<br>▪ ***Job category***<br>▪ ***Position title***<br>▪ ***Shift***<br>▪ ***Unit***	***Patient ID***<br>***OACIS***<br>▪ *Birth date*<br>▪ *Sex*	***Unit ID***<br>***ADT***<br>▪ *Unit category (inpatient/clinical/ outpatient)*<br>▪ *Unit type*<br>▪ *Unit name*<br>▪ *Establishment ID*<br>▪ *Date*<br>▪ *Total number of beds*<br>▪ *List of positions*<br>▪ *List of held positions*<br>▪ *Total number of patients admitted*<br>▪ *Total number of patients discharged*
Services dispensed<br>***HRM***<br>▪ ***Date of service***<br>▪ ***Work shift***<br>▪ ***Number of regular working hours paid***<br>▪ ***Number of overtime hours paid***	***Admission (Hospital stay)***<br>***ADT, OACIS***<br>▪ *Admission date*<br>▪ *Discharge date*<br>▪ *Medication*<br>▪ *Comorbidity*<br>▪ *Reason for admission*<br>▪ *Bed occupied (room no.)*<br>▪ *Unit*	
	Adverse events<br>***SISSS***<br>▪ *Name of adverse event*<br>▪ *Date (hour) of onset*<br>▪ *Occurrence circuit*<br>▪ *Medication incriminated*	
